# Association of alcohol use with memory decline in middle-aged and older Chinese: a longitudinal cohort study

**DOI:** 10.1186/s12888-022-04298-z

**Published:** 2022-11-01

**Authors:** Yu Meng Tian, Wei Sen Zhang, Chao Qiang Jiang, Feng Zhu, Ya Li Jin, Tong Zhu, Kar Keung Cheng, Lin Xu

**Affiliations:** 1 School of Public Health, Sun Yat-sen University, 74 Zhongshan 2nd Road, Guangzhou, Guangdong Province China; 2 Guangzhou Twelfth People’s Hospital, 510620 Guangzhou, China; 3 School of Public Health, the University of Hong Kong, Hong Kong, China; 4 Institute of Applied Health Research, University of Birmingham, Birmingham, UK

**Keywords:** Alcohol, Memory function, Cognition, Longitudinal, Elderly, Chinese

## Abstract

**Background:**

Previous studies on associations of alcohol use with memory decline showed inconclusive results. We examined these associations using longitudinal data from the Guangzhou Biobank Cohort Study (GBCS) and explored whether these associations varied by sex and age group.

**Methods:**

Memory function was assessed by delayed 10-word recall test (DWRT) and immediate 10-word recall test (IWRT) at both baseline (2003–2008) and follow-up (2008–2012) examinations, expressed as the mean annual change and mean annual rate of change in scores. Memory cognitive impairment was defined by DWRT scores of less than 4. Multivariable linear regression models and restricted cubic spline were used for data analysis.

**Results:**

Of 14,827 participants without memory cognitive impairment at baseline, 90.2% were never or occasional drinkers, 5% moderate drinkers, 1.5% excessive drinkers, and 3.3% former drinkers. The mean (standard deviation) age was 60.6 (6.6) years old. During an average of 4.1 years follow-up, 1000 (6.7%) participants developed memory cognitive impairment. After adjusting for confounders, compared with never or occasional drinkers, moderate and excessive drinkers had significant decline in DWRT scores (*β*, 95% confidence interval (*CI*) = -0.04 (-0.08 to -0.01), and − 0.07 (-0.14 to 0.01), respectively), and IWRT scores (*β*, 95% *CI* = -0.10 (-0.19 to -0.01), and − 0.15 (-0.30 to 0.01), respectively) annually. With respect to the mean annual rate of change, moderate and excessive drinkers also showed greater decline in DWRT scores (*β*, 95% *CI* = -1.02% (-1.87% to -0.16%), and − 1.64% (-3.14% to -0.14%), respectively). The associations did not vary by sex and age group (all P values for interaction ≥ 0.10).

**Conclusion:**

Compared to never or occasional alcohol use, moderate and excessive alcohol users had greater memory decline and the associations did not vary by sex and age group.

**Supplementary Information:**

The online version contains supplementary material available at 10.1186/s12888-022-04298-z.

## Introduction

Cognitive decline is one of the earliest preclinical symptoms of Alzheimer’s disease (AD) [1]. Of the domains of cognitive decline, memory decline is a critical feature [2, 3] and a very common complaint among the elderly. Impaired memory may severely affect daily functioning and mental health, and then reduce the quality of life. Hence, investigating this cognitive domain should be a priority. Previous studies showed a J- or U-shaped association between alcohol use and cognitive decline, suggesting that moderate alcohol use may protect cognitive function and reduce the risk of or delay the onset of dementia, whilst heavy alcohol use may increase the risk [4–6]. However, other studies showed that even a moderate amount of alcohol use, versus non-use, was associated with a higher risk of AD, suggesting a linear association between alcohol use and cognitive decline [7–9]. As the type of alcoholic beverage, ethnicity of the samples, and measurement methods of alcohol consumption and cognitive function differed across studies, whether alcohol use is associated with memory decline as well as the pattern of the association (i.e., J-/U-shaped or linear) is inconclusive and yet to be examined. Notably, our Mendelian Randomization study found no evidence of benefit from alcohol use at all levels of self-reported consumption [10]. As alcohol use is a modifiable behavior, scientific evidence concerning its safe use is of public health significance. However, due to conflicting or complex information about the protective or detrimental effects of alcohol [9, 11], as well as the long history and culture of alcohol drinking in China and western countries, alcohol control remains a complex area that requires more local evidence to support the decision.

Therefore, to provide more scientific evidence and support for alcohol control, we examined the association of alcohol use with longitudinal changes in memory performance in a population-based prospective cohort of middle-aged and older Chinese, taking into account various potential confounding factors.

## Methods

### Study sample and setting

The Guangzhou Medical Ethics Committee of the Chinese Medical Association approved the study and all participants gave written, informed consent before participation. Details of the Guangzhou Biobank Cohort Study (GBCS) have been reported previously [12, 13]. Briefly, the GBCS is a 3-way collaboration between Guangzhou Twelfth People’s Hospital and the Universities of Hong Kong and Birmingham. Participants were recruited from “The Guangzhou Health and Happiness Association for the Respectable Elders” (GHHARE), a community social and welfare organization, from September 2003 to January 2008. The first follow-up of participants was from March 2008 to December 2012. GHHARE is unofficially aligned with the municipal government. Membership is open to Guangzhou permanent residents aged 50 years or above for a nominal fee of 4 CNY (≈ 50 US cents) per month. GHHARE included about 7% of Guangzhou residents in this age group, with branches in all districts of Guangzhou, the capital city of Guangdong Province in Southern China. The baseline examination included a face-to-face computer-assisted interview by trained nurses to collect information on demographic characteristics, lifestyle, family, and personal medical history. Anthropometric parameters, blood pressure, fasting plasma glucose, lipids, and inflammatory markers were measured. Follow-up examination used interviews and clinical and laboratory examinations as the baseline. The reliability of the questionnaire was tested by recalling 200 randomly selected participants for re-interview and the results were satisfactory [12]. In the current study, 18,129 participants of the GBCS who had both baseline and follow-up assessments of memory recall were included. Of them, after excluding those with missing information and infeasible quantities of alcohol use (N = 146), missing data on baseline (N = 553) and follow-up delayed 10-word recall test scores (N = 467), and those with memory cognitive impairment at baseline (N = 2,136), 14,827 participants were included in the current study. The average follow-up period was 4.1 years.

### Exposures

Information on alcohol use was collected from baseline (2003–2008). Alcohol use status was classified into never, occasional, moderate, and excessive use based on the usual frequency of intake and the usual amount per occasion, which was described in our previous papers [14, 15]. The usual frequency and quantity of alcohol use of four beverage types: beer, grape wine, spirit, and Chinese rice wine, were assessed. Never drinkers were those who did not drink any alcoholic beverage throughout their life. Occasional drinkers were those who drank less than once per week, or drank only on special occasions, such as wedding parties or festivals, in the past 12 months. On average, the amount of alcohol use per week was almost zero and it is biologically unlikely to have any effect, for example, the incidence of dementia or other diseases [8, 16], due to occasional drinking. So we combined never and occasional drinkers into one group in data analysis. Moderate drinkers were people who drank at least once per week with less than or equal to 140 g (10 drinks) of ethanol for women and 210 g (15 drinks) of ethanol for men. Excessive drinkers were those who weekly drank more than 140 g (10 drinks) of ethanol in women and 210 g (15 drinks) of ethanol in men. Participants who had abstained from alcohol for at least one year were treated as former [17].

### Outcomes

Delayed and immediate memory recall was assessed by delayed 10-word recall test (DWRT) and immediate 10-word recall test (IWRT), respectively, and all participants were examined at baseline (2003–2008) and the first follow-up (2008–2012). Of these ten words, four were retained from the original English language test: “arm,” “ticket,” “grass,” and “letter” [18]. “Cabin,” “engine,” “pole,” and “shore” were substituted by “book,” “stick,” “corner,” and “stone” as in the adapted Consortium 10-word list learning task [19]. Meanwhile, to be more in tune with Chinese culture, “butter” and “queen” were replaced with “soy sauce” and “chairman.” During the first phase, the improved 10 words were read out to participants one by one and then they were immediately asked to recall the words. This procedure was repeated three times. The first three recalls were IWRT out of 30. After five minutes of answering other questions for distraction, participants were asked to recall as many words as they could remember. The last recall was DWRT out of 10. Participants were given one point for each word that they could be successfully recalled. The total number of correct words were recorded in IWRT and DWRT scores, respectively. The improved 10-word recall test was straightforward as well as time-saving and has been testified as a sensitive and efficient tool for dementia screening (mainly memory function) in developing countries [20]. Mean annual change and mean annual rate of change in both IWRT and DWRT scores were calculated. For example, mean annual change in IWRT scores = (follow-up scores – baseline scores) / follow-up time, mean annual rate of change in IWRT scores = (mean annual change / baseline scores) *100. A similar calculation was in the DWRT scores. Memory cognitive impairment was defined by DWRT scores of less than 4, corresponding to one standard deviation (SD) below the mean (mean ± SD: 5.5 ± 1.8) [21, 22].

### Potential confounders

To examine the extent to which potential factors explained the association of alcohol use with memory decline, we included two models. Model 1 was adjusted for sociodemographic factors, biological factors, lifestyle factors and self-rate health (poor/very poor). The fully adjusted model (model 2) was adjusted for sixteen factors: model 1 plus cardiovascular disease, hyperlipidemia, hypertension and type 2 diabetes. Sociodemographic factors included sex, age, education (secondary or below, college or above), occupation (manual, non-manual, others), marital status (married, others) and family income (< 10,000 CNY/year, 10,000–29,999 CNY/year, 30,000–49,999 CNY/year, ≥ 50,000 CNY/year, unknown). Biological factors included waist-to-hip-ratio, body mass index (BMI) and baseline DWRT/IWRT scores. Lifestyle factors included smoking status (never, former, current) and physical activity (inactive, minimally active, active). Physical activity was assessed by the International Physical Activity Questionnaire [23].

### Statistical analysis

For comparison of baseline characteristics by alcohol use status, we used the chi-squared test for categorical variables and one-way analysis of variance (ANOVA) for continuous variables. Multivariable linear regression models were used to assess the association of alcohol use with memory decline, reporting regression coefficient (*β*) and 95% confidence interval (*CI*). Considering the difference in drinking patterns and lifestyles between men and women, we assessed the association of alcohol use with memory decline stratified by gender and tested the interaction between alcohol use and sex. We also added the tests of interaction of alcohol use with age group. Moreover, we used restricted cubic spline analysis to assess the dose-response relationship between alcohol use and memory decline and the risk of memory cognitive impairment. P value < 0.05 was considered significant. Data analysis was performed using STATA/SE 16.0 (Stata Corp LP, College Station, TX, USA).

## Results

Of the 14,827 participants, the mean age was 60.6 years (standard deviation 6.6), and 73% participants were women. During an average follow-up of 4.1 years, 1000 (6.7%) participants developed memory cognitive impairment. Table 1 shows that 90.2% of participants were never or occasional alcohol drinkers, 5.0% moderate drinkers, 1.5% excessive drinkers and 3.3% former drinkers. In the current drinkers, the average amount of ethanol consumption was 23.74 g per day. Most never or occasional drinkers were women and alcohol drinkers were more likely to be men. Compared to never or occasional drinkers, moderate drinkers were older, had lower family income, lower proportion of being manual workers and self-rated poor health, and had a lower prevalence of cardiovascular disease, hypertension and type 2 diabetes, but had a higher proportion of current smokers, being married, levels of physical activity and waist-to-hip-ratio (all P < 0.05). No association of alcohol use with education levels, BMI or prevalence of hyperlipidemia was found (P from 0.11 to 0.41). Similar patterns were observed in excessive drinkers. Moreover, no differences in baseline DWRT scores by alcohol use was found (P = 0.48) and baseline IWRT scores varied between alcohol use groups (P < 0.001).

**Table 1:** Baseline characteristics of participants by alcohol use, Guangzhou Biobank Cohort Study, 2003–2008

	Alcohol use				P value
	**Never/occasional**	**Moderate**	**Excessive**	**Former**	
Number (%)	13,374 (90.2)	742 (5.0)	223 (1.5)	488 (3.3)	
Age, mean (SD), year	60.5 (6.6)	62.6 (6.8)	62.6 (6.4)	60.8 (6.7)	< 0.001
Sex, N (%)					
Men	3104 (23.2)	465 (62.7)	206 (92.4)	212 (43.4)	< 0.001
Women	10,270 (76.8)	277 (37.3)	17 (7.6)	276 (56.6)	
Education, N (%)					
Secondary or below	8554 (64.0)	459 (61.9)	158 (70.8)	313 (64.1)	0.27
College or above	4816 (36.0)	283 (38.1)	65 (29.2)	175 (35.9)	
Occupation, N (%)					
Manual	7817 (58.9)	389 (52.9)	130 (58.6)	269 (55.2)	< 0.001
Non-manual	3417 (25.7)	240 (32.6)	63 (28.4)	117 (24.0)	
Others	2047 (15.4)	107 (14.5)	29 (13.1)	101 (20.7)	
Marital status, N (%)					
Married	7487 (83.1)	440 (85.8)	150 (91.5)	300 (84.0)	0.02
Others	1522 (16.9)	73 (14.2)	14 (8.5)	57 (16.0)	
Smoking status, N (%)					
Never	11,490 (86.0)	403 (54.3)	35 (15.7)	322 (66.0)	< 0.001
Former	935 (7.0)	148 (20.0)	66 (29.6)	86 (17.6)	
Current	944 (7.0)	191 (25.7)	122 (54.7)	80 (16.4)	
Self-rated health, poor/very poor, N (%)	2073 (15.8)	97 (13.4)	19 (8.8)	72 (15.2)	0.01
Family income, CNY/year, N (%)					
<10,000	554 (4.2)	27 (3.6)	17 (7.6)	28 (5.7)	0.02
10,000–29,999	4202 (31.4)	225 (30.4)	81 (36.3)	167 (34.2)	
30,000–49,999	3160 (23.7)	166 (22.4)	43 (19.3)	124 (25.4)	
≥50,000	2617 (19.6)	150 (20.2)	42 (18.8)	89 (18.2)	
Unknown	2831 (21.2)	173 (23.4)	40 (17.9)	80 (16.4)	
Physical activity					
Inactive	1083 (8.1)	53 (7.1)	17 (7.6)	17 (3.5)	0.01
Minimally active	5271 (39.4)	291 (39.2)	88 (39.5)	185 (37.9)	
Active	7020 (52.3)	398 (53.6)	118 (52.9)	286 (58.6)	
BMI, mean (SD), kg/m^2^	23.8 (3.2)	23.7 (3.2)	23.8 (3.2)	24.0 (3.2)	0.41
Waist-to-hip-ratio, mean (SD)	0.86 (0.07)	0.88 (0.07)	0.91 (0.06)	0.87 (0.07)	0.03
Cardiovascular disease, yes, N (%)	5397 (40.4)	280 (37.8)	69 (31.1)	216 (44.4)	0.004
Hyperlipidemia, yes, N (%)	1514 (11.3)	69 (9.3)	18 (8.1)	47 (9.7)	0.11
Hypertension, yes, N (%)	3479 (26.0)	169 (22.8)	47 (21.2)	148 (30.5)	0.01
Type 2 diabetes, yes, N (%)	960 (7.2)	39 (5.3)	4 (1.8)	37 (7.6)	< 0.001
DWRT scores, mean (SD)	6.1 (1.5)	6.1 (1.5)	6.0 (1.6)	6.1 (1.5)	0.48
IWRT scores, mean (SD)	17.1 (3.6)	16.8 (3.5)	16.1 (3.4)	17.4 (3.6)	< 0.001

N = number; SD = standard deviation; BMI = body mass index; DWRT = delayed 10-word recall test; IWRT = immediate 10-word recall test

Table 2 shows that, after adjusting for sex, age, baseline DWRT scores, education, occupation, marital status, smoking, self-rated health, family income, physical activity, body mass index, waist-to-hip ratio, self-reported cardiovascular disease, hyperlipidemia, hypertension and type 2 diabetes, compared to never or occasional drinkers, greater decline in the mean annual change in DWRT scores was found in moderate drinkers and excessive drinkers (adjusted *β* (95% *CI*) = -0.04 (-0.08 to -0.01) and − 0.07 (-0.14 to 0.01), respectively). Similar patterns were observed using the annual rate of change in DWRT scores. No sex interaction on the changes in DWRT scores was found (all P values for sex-interaction ≥ 0.10).

**Table 2:** Changes in the delayed 10-word recall test (DWRT) scores during an average follow-up of 4.1 years by baseline alcohol use

		Alcohol use								
		**Never/occasional**			**Moderate**		**Excessive**		**Former**	
**Total**										
Mean annual change^#^										
Crude *β* (95% *CI*)	0.00		-0.08 (-0.12, -0.05)^***^			-0.14 (-0.20, -0.07)^***^		-0.03 (-0.07, 0.01)		
Model 1^a^	0.00		-0.04 (-0.08, -0.01)^*^			-0.06 (-0.14, 0.01)		-0.02 (-0.07, 0.03)		
Model 2^b^	0.00		-0.04 (-0.08, -0.01)^*^			-0.07 (-0.14, 0.01)		-0.03 (-0.08, 0.02)		
Mean annual rate of change^#^, %										
Crude *β* (95% *CI*)	0.00		-1.37 (-2.10, -0.65)^***^			-2.06 (-3.35, -0.77)^***^		-0.58 (-1.46, 0.30)		
Model 1^a^	0.00		-1.04 (-1.90, -0.19)^*^			-1.56 (-3.07, -0.05)^*^		-0.67 (-1.67, 0.33)		
Model 2^b^	0.00		-1.02 (-1.87, -0.16)^*^			-1.64 (-3.14, -0.14)^*^		-0.69 (-1.69, 0.31)		
**Men**										
Mean annual change										
Crude *β* (95% *CI*)	0.00			-0.10 (-0.15, -0.05)^***^		-0.10 (-0.18, -0.03)^**^		-0.02 (-0.09, 0.05)		
Model 1^a^	0.00			-0.08 (-0.13, -0.02)^**^		-0.05 (-0.13, 0.02)		-0.02 (-0.10, 0.06)		
Model 2^b^	0.00			-0.07 (-0.13, -0.02)^**^		-0.05 (-0.13, 0.02)		-0.03 (-0.11, 0.05)		
Mean annual rate of change, %										
Crude *β* (95% *CI*)	0.00			-1.58 (-2.53, -0.63)^**^		-1.68 (-3.05, -0.31)^*^		-0.22 (-1.57, 1.13)		
Model 1^a^	0.00			-1.79 (-2.92, -0.65)^**^		-1.23 (-2.83, 0.37)		-0.06 (-1.75, 1.63)		
Model 2^b^	0.00			-1.70 (-2.83, -0.58)^**^		-1.26 (-2.84, 0.33)		-0.07 (-1.77, 1.63)		
**Women**										
Mean annual change										
Crude *β* (95% *CI*)	0.00			-0.02 (-0.09, 0.04)		-0.05 (-0.31, 0.21)		-0.05 (-0.11, 0.02)		
Model 1^a^	0.00			-0.01 (-0.06, 0.06)		-0.28 (-0.53, -0.02)^*^		-0.03 (-0.09, 0.04)		
Model 2^b^	0.00			-0.01 (-0.06, 0.06)		-0.28 (-0.53, -0.03)^*^		-0.03 (-0.09, 0.03)		
Mean annual rate of change, %										
Crude *β* (95% *CI*)	0.00			-0.36 (-1.52, 0.81)		-0.88 (-5.53, 3.76)		-0.63 (-1.80, 0.53)		
Model 1^a^	0.00			0.05 (-1.28, 1.38)		-4.99 (-10.20, 0.21)		-0.98 (-2.23, 0.26)		
Model 2^b^	0.00			0.05 (-1.28, 1.37)		-5.02 (-10.23, 0.18)		-1.00 (-2.25, 0.24)		

^a^: Model 1: adjusted for sex, age, baseline DWRT scores, body mass index, waist-to-hip-ratio, education, occupation, marital status, smoking, self-rated health, family income and physical activity ^b^: Model 2: additionally adjusted for self-reported cardiovascular disease, hyperlipidemia, hypertension and type 2 diabetes ^#^: P values for interaction between sex and alcohol use were all 0.11 on the changes of DWRT scores^*^: P < 0.05; ^**^; P < 0.01; ^***^; P < 0.001

Table 3 shows that, after adjusting for the same confounders as in Table 2, compared with never or occasional drinkers, a significant decline in IWRT scores was also observed in moderate (*β*, 95% *CI* = -0.10, -0.19 to -0.01), former (-0.11, -0.21 to -0.01), and excessive drinkers (-0.15, -0.30 to 0.01). Although no sex interaction on the changes of IWRT scores was found (all P values for sex-interaction ≥ 0.10), the patterns by sex appeared to be slightly different. The mean annual change in IWRT scores appeared to have a greater decline in male moderate drinkers (*β* = -0.16, 95% *CI* -0.27 to -0.04), and the associations of moderate and excessive alcohol use with IWRT scores became non-significant in female drinkers. Moreover, the mean annual rate of decline in DWRT scores appeared to be more severe than IWRT scores both in moderate and excessive drinkers.

**Table 3:** Changes in the immediate 10-word recall (IWRT) scores during an average follow-up of 4.1 years by baseline alcohol use

	Alcohol use			
	**Never/occasional**	**Moderate**	**Excessive**	**Former**
**Total**				
Mean annual change^#^				
Crude *β* (95% *CI*)	0.00	-0.12 (-0.20, -0.04)^**^	-0.08 (-0.22, 0.07)	-0.16 (-0.27, -0.06)^**^
Model 1^a^	0.00	-0.10 (-0.19, -0.01)^*^	-0.14 (-0.29, 0.02)	-0.10 (-0.20, 0.01)
Model 2^b^	0.00	-0.10 (-0.19, -0.01)^*^	-0.15 (-0.30, 0.01)	-0.11 (-0.21, -0.01)^*^
Mean annual rate of change^#^, %				
Crude *β* (95% *CI*)	0.00	-0.68 (-1.21, 0.15)^*^	-0.51 (-1.47, 0.44)	-1.05 (-1.70, -0.40)^**^
Model 1^a^	0.00	-0.54 (-1.21, 0.14)	-0.75 (-1.93, 0.43)	-1.41 (-2.19, -0.63)^***^
Model 2^b^	0.00	-0.56 (-1.24, 0.11)	-0.82 (-2.00, 0.36)	-1.46 (-2.24, -0.67)^***^
**Men**				
Mean annual change				
Crude *β* (95% *CI*)	0.00	-0.14 (-0.25, -0.04)^**^	-0.02 (-0.18, 0.13)	-0.08 (-0.23, 0.07)
Model 1^a^	0.00	-0.16 (-0.27, -0.05)^**^	-0.14 (-0.30, 0.02)	-0.05 (-0.22, 0.12)
Model 2^b^	0.00	-0.16 (-0.27, -0.04)^**^	-0.14 (-0.30, 0.02)	-0.08 (-0.25, 0.09)
Mean annual rate of change, %				
Crude *β* (95% *CI*)	0.00	-0.95 (-1.64, -0.25)^**^	-0.26 (-1.27, 0.75)	-0.50 (-1.49, 0.49)
Model 1^a^	0.00	-1.26 (-2.14, -0.38)^**^	-0.74 (-1.99, 0.50)	-0.95 (-2.27, 0.36)
Model 2^b^	0.00	-1.26 (-2.15, -0.38)^**^	-0.76 (-2.01, 0.49)	-1.03 (-2.36, 0.30)
**Women**				
Mean annual change				
Crude *β* (95% *CI*)	0.00	-0.01 (-0.15, 0.13)	-0.07 (-0.61, 0.46)	-0.20 (-0.34, -0.07)^**^
Model 1^a^	0.00	-0.01 (-0.15, 0.13)	-0.31 (-0.85, 0.23)	-0.12 (-0.25, 0.01)
Model 2^b^	0.00	-0.01 (-0.15, 0.13)	-0.32 (-0.86, 0.22)	-0.13 (-0.26, -0.01)^*^
Mean annual rate of change, %				
Crude *β* (95% *CI*)	0.00	0.19 (-0.67, 1.06)	0.06 (-3.37, 3.49)	-1.33 (-2.19, -0.47)^*^
Model 1^a^	0.00	0.58 (-0.47, -1.63)	-1.67 (-5.76, 2.41)	-1.65 (-2.63, -0.67)^*^
Model 2^b^	0.00	0.53 (-0.51, 1.59)	-1.73 (-5.81, 2.36)	-1.68 (-2.65, -0.70)^*^

^a^: Model 1: adjusted for sex, age, baseline IWRT scores, body mass index, waist-to-hip-ratio, education, occupation, marital status, smoking, self-rated health, family income and physical activity ^b^: Model 2: additionally adjusted for self-reported cardiovascular disease, hyperlipidemia, hypertension and type 2 diabetes^#^: P values for interaction between sex and alcohol use were 0.28 and 0.11 on the changes of IWRT scores ^*^: P < 0.05; ^**^: P < 0.01; ^***^: P < 0.001

**Fig. 1:**
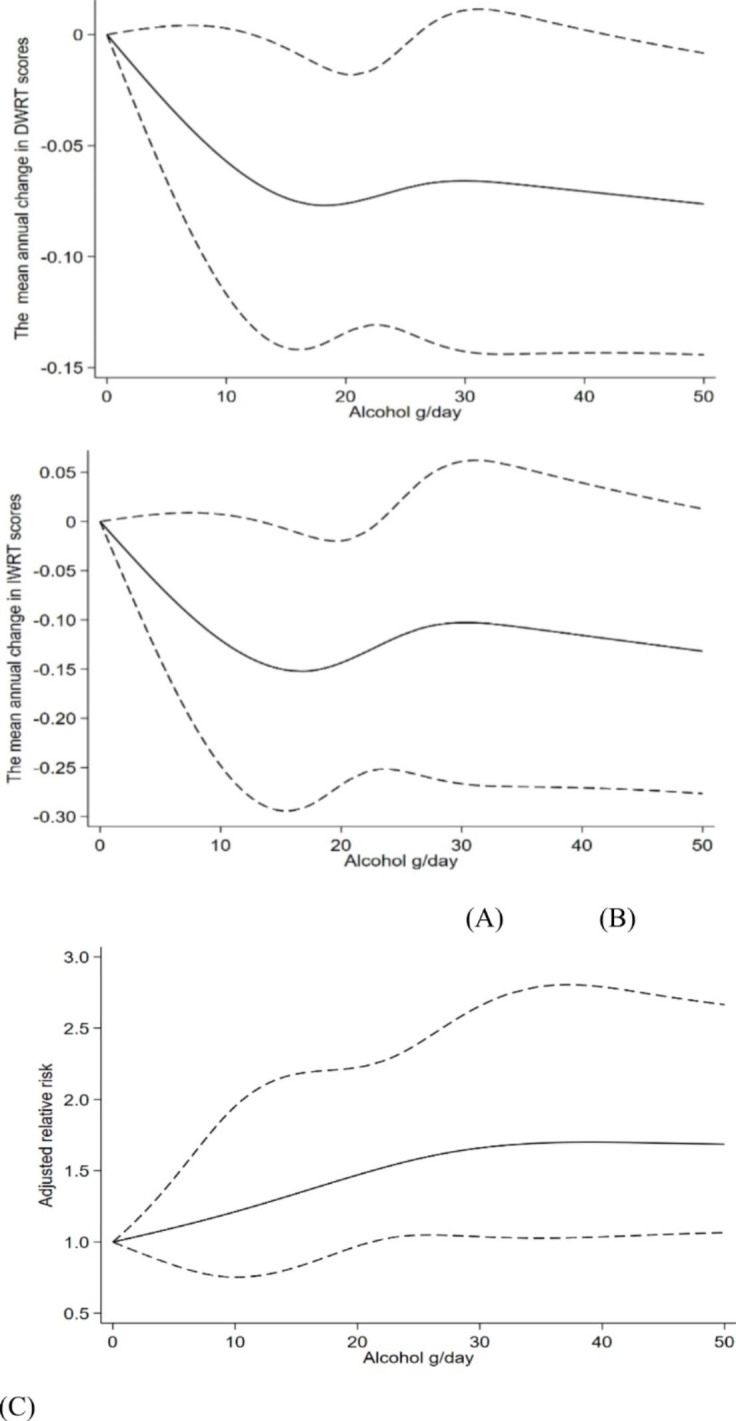
Association of daily alcohol use (g/day) with the mean annual change in DWRT scores (A), IWRT scores (B) and the risk of memory cognitive impairment (C) during an average follow-up of 4.1 years Note: [1] *β* (solid line) and 95% confidence intervals (dashed line) and relative risk (solid line) and 95% confidence intervals (dashed line) were adjusted for sex, age, baseline DWRT/IWRT scores, body mass index, waist-to-hip-ratio, education, occupation, marital status, smoking, self-rated health, family income, physical activity, self-reported cardiovascular disease, hyperlipidemia, hypertension and type 2diabetes. [2] P values for non-linearity from 0.20 to 0.96

Figure 1 shows the more alcohol consumed, the greater decline in DWRT and IWRT scores, and the higher risk of memory cognitive impairment after adjusting for potential confounders (P values for non-linearity from 0.20 to 0.96).

Sensitivity analysis shows no evidence that the associations of alcohol use with the mean annual change and mean annual rate of change in DWRT/IWRT scores and the risk of memory cognitive impairment varied by age group (P values for interaction from 0.25 to 0.94) (TableS1).

## Discussion

In this population-based longitudinal study of older adults, we found that compared with never or occasional alcohol use, moderate and excessive alcohol users had a faster decline in delayed and immediate recall memory scores and the associations did not vary by sex and age group. Our study extends the previous findings to a population with a relatively restrained pattern of alcohol consumption. And World Health Organization (WHO) pointed out that lifetime abstention from alcohol drinkers were most prevalent in Asia [24], which explained why our sample had fewer moderate and excessive alcohol drinkers than other studies especially those conducted in western countries. As such our study did not support the U/J-shaped association between alcohol use and cognitive function. Although we cannot draw firm conclusions from an observational study, our results suggest that it is unlikely that moderate alcohol use has a beneficial effect on cognitive function, which was in line with our previous Mendelian randomization study [10]. Regarding the increasing amount of alcohol use among middle-aged and older people in the past decades, our findings add to the evidence on the detrimental effects of alcohol use [25, 26], which can be used to inform public health strategies on alcohol control. Furthermore, our findings indicate that efforts in reducing alcohol consumption and the associated health burden need to be enforced. Middle-aged to older adults are encouraged to reduce, or quit alcohol consumption to protect cognitive function.

### Comparison with previous studies

Our results observed moderate and excessive alcohol drinkers had a faster decline in memory function. And some previous studies also found these associations in different races and proportions of alcohol drinkers [27–29]. A prospective cohort study in the US with 6339 participants showed that among women, moderate alcohol drinkers (< 28 g/day) had poor performance in the aspect of memory function [27]. Another observational cohort study in the UK of 550 participants found those drinking moderately (20–30 g/day) and excessively (> 43 g/day) had memory decline, probably through the changes in hippocampal atrophy [28]. However, the protective association of moderate alcohol use with cognitive function was observed in some studies [30–33]. There are several possible reasons for the discrepancy between our findings and the protective effects of moderate alcohol use seen in the West. It may be that in East Asian populations such as ours, observational studies on alcohol use are less biased by changes in alcohol use with ill-health and social desirability bias, particularly in men, in whom alcohol use is accepted but optional. Alcohol consumption is a common social activity in the West. People who are more health-conscious and self-control tend to drink moderately than other people (i.e., healthy moderate drinker effect). Moreover, this moderation not only presented in alcohol use but also in many aspects, for example, physical activity and dietary pattern [34]. Thus the favorable effect on cognitive function could be explained by the healthy behaviors rather than moderate alcohol use per se. Furthermore, other studies draw unrelated conclusions. Two meta-analyses found no association between moderate alcohol use and cognitive impairment, with a higher degree of heterogeneity [35, 36]. And some observational studies also showed such association [37–39], which could be due to insufficient sample size, inadequate reference group used, or outcome measures. For example, a cross-sectional study of sixty-six adults reported that compared to nondrinkers, excessive alcohol use (> 28 g/day for men and > 14 g/day for women) was significantly associated with memory impairment, whereas no association for moderate alcohol use was found [40]. Similarly, the Whitehall II cohort found that heavy (≥ 36 g/day) but not moderate (20 ~ 35 g/day) drinking in men was associated with a faster decline in short-term memory compared with men who drank < 20 g per day [41]. Another large prospective cohort study of eastern Europeans showed no association of moderate alcohol use, versus light drinkers (< 10 g/day for men and < 5 g/day for women) with delayed or immediate memory recall [42]. However, this study assessed the association of alcohol consumption with baseline or follow-up cognitive performance but not the rate of cognitive decline. In addition, results of cross-sectional studies in Asians also showed inconsistent results [43, 44]. One study of 585 elderly Japanese men showed no association between alcohol consumption and memory function [43]. Another study of 16,328 Chinese using baseline data of the China Health and Retirement Longitudinal Study (CHRLS) showed that moderate alcohol use (≤ 28 g/day) was not associated with memory function, whereas at-risk (> 28 g/day) drinkers had lower scores of memory performance than never drinkers. Regarding the inconsistent findings of the previous studies, our study adds to the literature by using longitudinal data to assess the association with cognitive decline, with the results generally consistent with the cross-sectional findings in terms of excessive alcohol use from the CHRLS [44].

### Mechanism

There are some possible mechanisms underlying the association between alcohol use and memory function. One explanation was alcohol induced oxidative stress and inflammatory responses in the hippocampus [45], which is closely related to memory function [46]. Oxidative stress refers to the cytopathologic consequences of a mismatch between the production of free radicals and the ability of the cell to defend against them, which leads to elevated intracellular levels of reactive oxygen species and subsequent damage to lipids, proteins, and deoxyribonucleic acid [47]. In the hippocampal cells of alcohol exposure, oxidative stress responses increased and antioxidant components such as lipid peroxidase, glutathione, and superoxide dismutase decreased rapidly [48]. On the other hand, alcohol exposure also induced neuroinflammation responses, triggering the production of pro-inflammatory cytokines such as tumor necrosis factor -α and interleukin-1β, and activating the microglia, and astrocytes in the hippocampus [45]. Moreover, the activated microglia can also produce reactive oxygen species and reduce the levels of intracellular glutathione [49].

### Strengths and limitations

The strengths of this study included the large sample size, population-based longitudinal study design with an adequate follow-up period, repeated examination of both immediate and delayed recall memory function, and comprehensive measurement of alcohol use and covariates. However, there were several limitations in our study. Firstly, as alcohol use was assessed by self-report, social-desirability bias could not be completely ruled out [50]. This might have led to an under-reporting of alcohol use, thus the strength of the association of excessive alcohol use with memory function could be underestimated. Secondly, due to the relatively small number of alcohol users in our study, we did not have sufficient participants to further breakdown by type of alcoholic beverage, although there is no evidence that the effects of ethanol on cognitive function vary by beverage type. In addition, the marginally non-significant results in excessive drinkers could also be due to insufficient statistical power. However, the tests for linearity on the associations of alcohol use status with changes in DWRT/IWRT scores showed suggestive evidence for linearity, indicating that excessive drinkers might have the greatest decline in DWRT/IWRT scores. Thirdly, the effects of binge drinking were not assessed because it was rare in middle-aged and older Chinese [51, 52]. Fourthly, the years of abstinence and previous alcohol use in former drinkers were not detailed. Further studies will focus on the association of abstinence with memory decline. Meanwhile, a few alcohol drinkers may abstain due to their own health conditions, which could also lead to an underestimation of the cognitive decline related to alcohol use. Fifthly, we did not examine the psychometric appropriateness of the adapted 10-word recall test, although this test has been used in many previous studies [10, 21, 53]. Future studies examining the psychometric appropriateness of this test in various populations are warranted. Finally, as patterns of alcohol use might vary across settings and all participants were recruited from a city in China, the generalizability of the results to other populations may be limited, although biologically there is no reason to think why it cannot be generalized.

## Conclusion

Compared with never or occasional alcohol use, both moderate and excessive alcohol users had greater memory decline and the associations did not vary by sex and age group. Moreover, the risk of memory cognitive impairment increased with the amount of alcohol use.

## Electronic supplementary material

Below is the link to the electronic supplementary material.

Supplementary Material 1. Changes in the DWRT and IWRT scores and memory cognitive impairment during an average follow-up of 4.1 years by baseline alcohol use and age group

## Data Availability

Ethical approval in place allows us to share data on requests. Please directly send such requests to the Guangzhou Biobank Cohort Study Data Access Committee (gbcsdata@hku.hk).

## Abbreviations

DWRT: Delayed 10-word recall test.
IWRT: Immediate 10-word recall test.
