# Supplementary material for: Association of alcohol use with memory decline in middle-aged and older Chinese: a longitudinal cohort study
Source: BMC Psychiatry. 2022 Nov 1;22:673. doi: 10.1186/s12888-022-04298-z (PMC9623936; doi:10.1186/s12888-022-04298-z)
Supplement: Supplementary file 1 — Supplementary Material 1. Changes in the DWRT and IWRT scores and memory cognitive impairment during an average follow-up of 4.1 years by baseline alcohol use and age group [file 12888_2022_4298_MOESM1_ESM.docx]

**Table S1.** Changes in the DWRT and IWRT scores and memory cognitive impairment during an average follow-up of 4.1 years by baseline alcohol use and age group

|  | **Alcohol use** | | | | |
| --- | --- | --- | --- | --- | --- |
|  | **Never/occasional** | **Moderate** | **Excessive** | **Former** | |
| **DWRT** | | | | | |
| **Age <65 years** | | | | | |
| Number of participants | 9803 | 464 | 145 | 346 |  |
| Mean annual change^§^ | | | | | |
| Crude *β* (95% *CI*) | 0.00 | -0.07 (-0.11, -0.02)^**^ | -0.17 (-0.24, -0.09)^***^ | -0.03 (-0.08, 0.02) | |
| Model 1^a^ | 0.00 | -0.03 (-0.08, 0.03) | -0.08 (-0.17, 0.01) | -0.02 (-0.08, 0.04) | |
| Model 2^b^ | 0.00 | -0.03 (-0.08, 0.03) | -0.08 (-0.17, 0.01) | -0.02 (-0.08, 0.04) | |
| Mean annual rate of change^§^, % | | | | | |
| Crude *β* (95% *CI*) | 0.00 | -1.17 (-2.06, -0.27)^*^ | -2.63 (-4.20, -1.05)^**^ | -0.70 (-1.74, 0.33) | |
| Model 1^a^ | 0.00 | -0.84 (-1.93, 0.26) | -1.58 (-3.41, 0.24) | -0.69 (-1.86, 0.47) | |
| Model 2^b^ | 0.00 | -0.83 (-1.93, 0.27) | -1.61 (-3.44, 0.21) | -0.71 (-1.88, 0.45) | |
| **Age ≥65 years** | | | | | |
| Number of participants | 3571 | 278 | 78 | 142 |  |
| Mean annual change^§^ | | | | | |
| Crude *β* (95% *CI*) | 0.00 | -0.06 (-0.12, -0.01)^*^ | -0.04 (-0.14, 0.07) | -0.02 (-0.11, 0.06) | |
| Model 1^a^ | 0.00 | -0.07 (-0.14, -0.01)^*^ | -0.06 (-0.19, 0.07) | -0.03 (-0.12, 0.07) | |
| Model 2^b^ | 0.00 | -0.07 (-0.14, -0.01)^*^ | -0.06 (-0.18, 0.07) | -0.03 (-0.12, 0.07) | |
| Mean annual rate of change^§^, % | | | | | |
| Crude *β* (95% *CI*) | 0.00 | -1.29 (-2.51, -0.07)^*^ | -0.65 (-2.90, 1.60) | -1.16 (-1.84, 1.52) | |
| Model 1^a^ | 0.00 | -1.26 (-2.64, 0.11) | -1.91 (-4.55, 0.73) | -0.57 (-2.50, 1.35) | |
| Model 2^b^ | 0.00 | -1.27 (-2.65, 0.10) | -1.87 (-4.51, 0.77) | -0.50 (-2.43, 1.44) | |
|  |  |  |  |  | |
| **IWRT** | | | | | |
| **Age <65 years** | | | | | |
| Mean annual change^§^ | | | | | |
| Crude *β* (95% *CI*) | 0.00 | -0.14 (-0.23, -0.05)^**^ | -0.26 (-0.42, -0.10)^**^ | -0.12 (-0.22, -0.01)^*^ | |
| Model 1^a^ | 0.00 | -0.08 (-0.20, 0.03) | -0.13 (-0.33, 0.06) | -0.06 (-0.18, 0.06) | |
| Model 2^b^ | 0.00 | -0.08 (-0.20, 0.03) | -0.14 (-0.33, 0.06) | -0.06 (-0.19, 0.06) | |
| Mean annual rate of change^§^, % | | | | | |
| Crude *β* (95% *CI*) | 0.00 | -0.47 (-1.14, 0.19) | -0.55 (-1.73, 0.61) | -1.30 (-2.06, -0.53)^**^ | |
| Model 1^a^ | 0.00 | -0.47 (-1.34, 0.41) | -0.52 (-1.97, 0.93) | -1.27 (-2.19, -0.35)^**^ | |
| Model 2^b^ | 0.00 | -0.46 (-1.33, 0.42) | -0.54 (-1.99, 0.91) | -1.30 (-2.22, -0.37)^**^ | |
| **Age ≥65 years** | | | | | |
| Mean annual change^§^ | | | | | |
| Crude *β* (95% *CI*) | 0.00 | -0.12 (-0.25, 0.02) | -0.02 (-0.26, 0.23) | -0.06 (-0.24, 0.12) | |
| Model 1^a^ | 0.00 | -0.13 (-0.26, 0.01) | -0.19 (-0.46, 0.06) | -0.18 (-0.37, 0.01) | |
| Model 2^b^ | 0.00 | -0.13 (-0.27, 0.01) | -0.19 (-0.45, 0.06) | -0.19 (-0.38, -0.01)^*^ | |
| Mean annual rate of change^§^, % | | | | | |
| Crude *β* (95% *CI*) | 0.00 | -0.76 (-1.66, 0.14) | -0.21 (-1.86, 1.43) | -0.38 (-1.61, 0.86) | |
| Model 1^a^ | 0.00 | -0.62 (-1.68, 0.44) | -1.41 (-3.44, 0.62) | -1.83 (-3.32, -0.35)^*^ | |
| Model 2^b^ | 0.00 | -0.65 (-1.71, 0.41) | -1.39 (-3.43, 0.65) | -1.80 (-3.30, -0.31)^*^ | |
|  |  |  |  |  | |
| **Memory cognitive impairment**^§^ | | | | | |
| **Age <65 years** | | | | | |
| Crude RR (95% *CI*) | 1.00 | 1.16 (0.78, 1.72) | 2.44 (1.46, 4.08)^**^ | 1.02 (0.59, 1.76) | |
| Model 1^a^ | 1.00 | 1.05 (0.60, 1.83) | 2.19 (1.09, 4.38)^*^ | 1.25 (0.64, 2.44) | |
| Model 2^b^ | 1.00 | 1.05 (0.61, 1.84) | 2.25 (1.12, 4.52)^*^ | 1.28 (0.66, 2.51) | |
| **Age ≥65 years** | | | | | |
| Crude RR (95% *CI*) | 1.00 | 1.23 (0.89, 1.72) | 1.22 (0.68, 2.16) | 1.42 (0.89, 2.24) | |
| Model 1^a^ | 1.00 | 1.24 (0.83, 1.87) | 1.22 (0.63, 2.37) | 1.24 (0.71, 2.19) | |
| Model 2^b^ | 1.00 | 1.25 (0.83, 1.88) | 1.21 (0.62, 2.36) | 1.23 (0.70, 2.16) | |

^a^: Model 1: adjusted for sex, age, baseline DWRT/IWRT scores, body mass index, waist-to-hip-ratio, education, occupation, marital status, smoking, self-rated health, family income and physical activity

^b^: Model 2: additionally adjusted for self-reported cardiovascular disease, hyperlipidemia, hypertension and type 2 diabetes

^§^: P values for interaction between age group and alcohol use from 0.25 to 0.94

^*^: P < 0.05; ^**^: P < 0.01; ^***^: P < 0.001
